# Caloric: Its Agencies in the Phenomena of Nature

**Published:** 1860-11

**Authors:** 


ART. V.—*Caloric: its Mechanical, Chemical, and Vital Agencies in the Phenomena of Nature.* By SAMUEL L. METCALFE, M.D., late of Transylvania University. 2 vols., 8vo., pp. 630 and 481. Philadelphia: J. B. Lippincott & Co., 1859.

IN a former number of the REVIEW* we took occasion to express our opinion of the merits and demerits of this remarkable work, which originally appeared in London in 1843, and was justly regarded, at the time, as a new and extraordinary system of natural philosophy, physiology, pathology, and therapeutics.

It is not our intention, at this late period, to rehearse the story of the author's enthusiastic fondness for the study of nature, of his wonderful power of generalization, of his bold and earnest attempt to reduce the phenomena of nature to a few comprehensive principles capable of explaining all that is involved and obscure in natural philosophy and biol-

* For September, 1858.

ogy, of the difficulties he experienced in making this attempt, of his pilgrimage to London, of his persevering studies there, and of the tedious birth of his book amid poverty and distress.

Neither is it our purpose to dwell upon the contents of these volumes. These were long ago laid before the profession in various able reviews, both critical and analytical, which appeared in the leading medical and scientific journals both of Great Britain and this country. By the editor of the London *Medical Gazette* the work was pronounced to be "the truest specimen and most successful achievement of that which the late Earl of Bridgewater had in view, when he left a sum of money for the purchase and publication of a series of treatises illustrative of the wisdom and goodness of God in creation." Other high encomiums were bestowed upon it in the medical periodicals of that time.

All this, however, belongs to the past and its endless chronicles of good and bad.

Our object in noticing the book at all, is to make a passing comment upon the new edition, which is simply a reprint of the original work, with the addition of a short appendix, written by B. H. R., the gentleman to whom was entrusted the editorial supervision.

We deeply regret that this work should have been given to the world again in its old form, with the date of 1859 upon the title-page, and with various imperfections of which the author was aware, and which it was his intention, had he lived, to have rectified in a new edition. When published in 1843, it was fully up to the times in the accuracy and reliability of its facts; this cannot be said of it, however, in its new dress of 1859, which is calculated rather to detract from his reputation than to increase it. In the sixteen years that intervene between the two editions, the aspect of science has been modified in various particulars. An unwearied student, Dr. Metcalfe kept himself thoroughly posted, up to the time of his death, upon all actual advancement in science. During the last thirteen years of his life, though he published nothing, he wrote a great deal; and, had his life been spared, he would have reproduced his book in a considerably modified form. The intelligent reader will see that the third chapter of this work is strictly fundamental, and that the whole theory of the author revolves around it as around a centre. If the generalizations contained in this chapter are legitimate, the correctness of the whole work must be admitted. If they are erroneous, the whole superstructure erected upon them falls to the ground, the baseless fabric of a dream. Six months of close study and experiment were consumed by the author in the production of this part of his system. It was his intention to have remodeled this chapter and to have introduced into it some newer views and still broader generalizations concerning motion as it is exhibited to us in both the organic and inorganic worlds. Some of his opinions upon this subject he committed to paper, examining and criticising freely the dynamical theory of heat and the facts and speculations brought forward by Joule and others, relative to its mechanical equivalent. In this article he also intended to examine at length the theorem of Laplace, concerning the state of aggregation of bodies; and the views of Graham upon the movement of liquids in the phenomena of endosmose and exosmose. It was his intention also, to have largely commented upon and in some respects to have severely criticised the doctrine of the correlation of the physical, and of the physical with the vital forces, as advocated by Grove, Carpenter, and others. We have in our possession a copy of the first edition of Grove's Essay, the margins of which are, in various places, filled with notes written in lead-pencil by Dr. Metcalfe, and expressing a different interpretation of the facts brought forward by Grove.

In turning over the pages of "Caloric," we observe several paragraphs, and even whole pages, for which the author intended to substitute others more in keeping with the present state of science. He several times informed us in conversation, that he intended to alter some of the chemical tables in the first volume, and introduce several others.

These statements we make with the desire to do justice to the memory of a deceased friend, whose scientific reputation is more likely to be impaired than benefited by the republication of his work in its present form.

